# Fe_3_O_4_-Filled Cellulose Paper for Triboelectric Nanogenerator Application

**DOI:** 10.3390/polym15010094

**Published:** 2022-12-26

**Authors:** Wimonsiri Yamklang, Teerayut Prada, Weeraya Bunriw, Walailak Kaeochana, Viyada Harnchana

**Affiliations:** 1 Department of Physics, Khon Kaen University, Khon Kaen 40002, Thailand; 2 Institute of Nanomaterials Research and Innovation for Energy (IN-RIE), Khon Kaen University, Khon Kaen 40002, Thailand

**Keywords:** cellulose paper, triboelectric nanogenerator, Fe_3_O_4_, sugarcane leaves

## Abstract

Cellulose-based materials have recently drawn much interest due to their sustainability, biodegradability, biocompatibility, and low cost. In this present work, cellulose fiber paper (CFP) was fabricated from sugarcane leaves and used as a friction material for a triboelectric nanogenerator (TENG). Fe_3_O_4_ was incorporated to CFP triboelectric material to increase the dielectric constant of CFP for boosting power generation of TENG. The Fe_3_O_4_ filled CFP was synthesized using a facile one-pot co-precipitation technique. The effect of Fe_3_O_4_ content in CFP on dielectric property and TENG performance was investigated and optimized. The CFP filled with Fe_3_O_4_ nanoparticles exhibited the improved dielectric constant and possessed a superior TENG performance than pristine CF. The highest power density of 1.9 W/m^2^ was achieved, which was able to charge commercial capacitors serving as a power source for small electronic devices.

## 1. Introduction

Triboelectric nanogenerator (TENG) has attracted growing interest in research as a new power generation technology that is clean and sustainable. With the advent of portable and wearable electronic devices, wireless and sensor networks, an effective and sustainable power source for those systems are required. TENG is emerging as potential solution that converts mechanical energy into electricity based on a combination of contact electrification and electrostatic induction effects [1]. TENG offers many appealing aspects such as high power output, simple fabrication with diverse choices of materials, and many operation modes, with potential for various applications including micro/nano power sources [2], self-powered sensors [3], air/gas filtration [4] and control interface [5,6]. 

Cellulose is the most abundant natural polymer, which is biodegradable and biocompatible as well as renewable. Since the main commercial product of cellulose is paper, many paper-based electronic devices are being explored for the development of next generation wearable and flexible electronics [7]. Paper-based TENG is therefore attractive as a component for TENG, which could be used for a wide range of applications such as a power source, sensing and actuating. Cellulose paper also offers many appealing advantages for TENG application including low cost, biodegradability and biocompatibility, ease of modification and good physical properties [8]. Recently, cellulose paper-based TENGs were reported [8,9,10,11], and the most exciting progress on the cellulose based TENG was proposed with the highest power density of 300 W/m^2^ [9].

As for enhancing TENG electrical output for practical applications, many strategies have been proposed for modifying cellulose-based TENG including surface physical modification [12,13], surface chemical modification [14], and material composition modulation [8,15]. Among these approaches, the incorporations of nanostructured materials were found to effectively improve the electrical output of cellulose-based TENG. In particular, filling cellulose with high dielectric constant nanoparticles such as BaTiO_3_ was found to not only increase surface roughness, but also increase the charge capacitance ability of cellulose material, resulting in the significant improvement of TENG output performance [10]. Fe_3_O_4_ or magnetite is one of the nanoparticle fillers widely used for improving dielectric properties of composite materials [16,17,18,19,20,21], which showed a high dielectric constant with a high breakdown field [18]. Fe_3_O_4_ were able to generate interfacial polarizations, which was one of the most effective approaches to improving dielectric constant in many triboelectric polymers in order to enhance TENG electrical outputs [22,23,24,25,26]. However, the incorporation of Fe_3_O_4_ in cellulose-based TENG has not been reported thus far.

In the present work, cellulose fiber paper (CFP) was fabricated from sugarcane leaves and employed as a friction layer for a TENG. Fe_3_O_4_ nanoparticles were synthesized using the co-precipitations technique. Taking advantage of the strong basicity condition in the cellulose fiber (CF) preparation process, Fe_3_O_4_ nanoparticles were able to precipitate in CF as a one-pot reaction. The obtained CF filled with Fe_3_O_4_ nanoparticles was then produced into CF papers, which were subsequently used as tribopositive materials for TENG. The effects of Fe_3_O_4_ nanoparticles at various concentrations in CFP paper on dielectric properties and TENG electrical output of the CFP-based TENGs were studied. This work has proposed the fabrication of green and sustainable TENG with high energy conversion performance, using natural-based material with simple fabrication and at low cost.

## 2. Materials and Methods

### 2.1. Materials and Chemicals

Sugarcane leaves (SL) were obtained from a local sugarcane farm, Buriram, Thailand. Sodium hydroxide (NaOH, 97%) was purchased from KEMAUS (New South Wales, Australia). FeCl_2_·4H_2_O and FeCl_3_·6H_2_O were purchased from SIGMA-ALDRICH (St. Louis, MO, USA). 2-Hydroxyethyl cellulose (HEC) was obtained from SIGMA-ALDRICH (St. Louis, MO, USA). Hydrogen peroxide (H_2_O_2_, 35%) was purchased from ANaPURE (Brightchem Sdn. Bhd., Selangor, Malaysia). Hydrochloric acid solution (HCl, 37%) was obtained from RCI Labscan (Bangkok, Thailand). 

### 2.2. Synthesis of Cellulose Paper with Fe Oxide Nanoparticle Fillers 

Cellulose fibers were extracted from SL, as described in our previous work [27]. Briefly, SL were firstly washed and dried in an oven at 60 °C for 48 h. Dried SL were cut and ground with a grinder machine to obtain powders the size of approximately 300 µm. The alkaline treatment was undertaken by mixing 30.0 g of SL powder with 10% NaOH at a solid/liquid ratio of 1:20 g/mL and heated at 90 °C for 4 h. After that, the mixture was filtered and washed with DI water until a neutral pH was obtained. The alkaline peroxide was used as a bleaching treatment, which was prepared from a mixture of 25% H_2_O_2_ and 2% NaOH solutions (at H_2_O_2_ to NaOH volume ratio of 4:1). The bleaching step was performed at 90 °C for 3 h. The product was then washed with DI water to reach a neutral pH, and the slurry of CF microfibers was obtained. 

To fabricate CF paper filled with Fe_3_O_4_ nanoparticles, the preparation detail is described as follows. FeCl_2_·4H_2_O (Fe(II)) and FeCl_3_·6H_2_O (Fe(III)) at various concentrations as listed in Table 1 were dissolved with 10 mL deionized water in the separated flask. The Fe precursor solutions were heated at the temperature of 90 °C and kept constant for 30 min. Then, the Fe precursors were added to the CF slurry and kept at 90 °C for 10 min. The 2.5 M NaOH was then added to the mixture to obtain pH of 12. The mixture was left stirred at 55 °C for 2 h and then let at room temperature for 24 h. The product was washed to neutrality with DI water. The CF with Fe_3_O_4_ nanoparticles was filtrated to prepare paper for TENG fabrication, as described in the following section. 

### 2.3. Preparation of Cellulose Paper Nanogenerator

4 g of the CF-Fe_3_O_4_ paste from Section 2.2 was mixed with 1.6 mL HEC solution (0.5 g in 10 mL). The paste was then cast on a 4 × 4 cm^2^ ITO substrate to obtain the thickness of approximately 1 mm. A set of three samples was prepared for each experimental condition. The samples were then dried at 60 °C for 12 h. The specimens were then tested for the energy conversion performance.

### 2.4. Material Characterizations

Morphologies and elemental compositions of the prepared specimens were examined by scanning electron microscope (SEM) (Helios Nanolab, FEI) equipped with energy dispersive x-ray analysis (EDX). Chemical structure analysis was performed using a Fourier-transform infrared spectroscopy (FTIR) (TENSOR27). Crystal structure of the specimens were probed by XRD (PANalytical EMPYREAN). Dielectric constants of the samples were measured by using a Keysight E4990A impedance analyzer at 10^2^–10^6^ Hz.

### 2.5. TENG Output Measurement

The prepared CFP on ITO substrates were used as tribopositve materials, and a Teflon sheet was used as a contact tribonegative material with the contact areas of 4 × 4 cm^2^. The electrical outputs were acquired with a single electrode operation mode under an impact force of 4 N at frequency of 5 Hz. Output voltage and current were measured using an oscilloscope (Tektronix DPO2002B) and a digital ammeter (Keithley DMM6500), respectively. 

## 3. Results and Discussion

**Figure 1:**
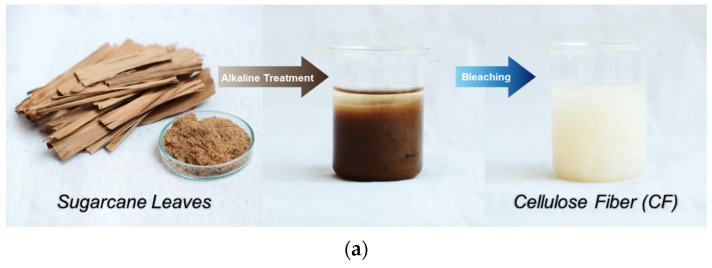

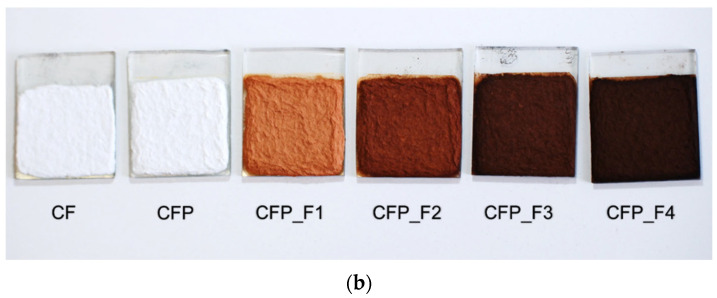
(**a**) The CF products after alkaline treatment and bleaching; (**b**) photographs of CF, CFP, CFP_F1-F4 on ITO substrates as tribopositive materials for TENG.

The fabrication process of the CF is illustrated in Figure 1a, displaying the physical appearances of alkaline treated and bleached SL products. The photographs of the prepared CF and CFP_F1-F4 TENGs coated on ITO glasses are presented in Figure 1b. The CF and CFP were white in color, whereas CF–Fe_3_O_4_ papers were brown and darker in shade with increasing Fe (II/III) precursors. The difference between CF and CFP is that CFP had an HEC additive as a binding agent, whereas CF contained only pristine cellulose fiber, with no HEC added. 

**Figure 2:**
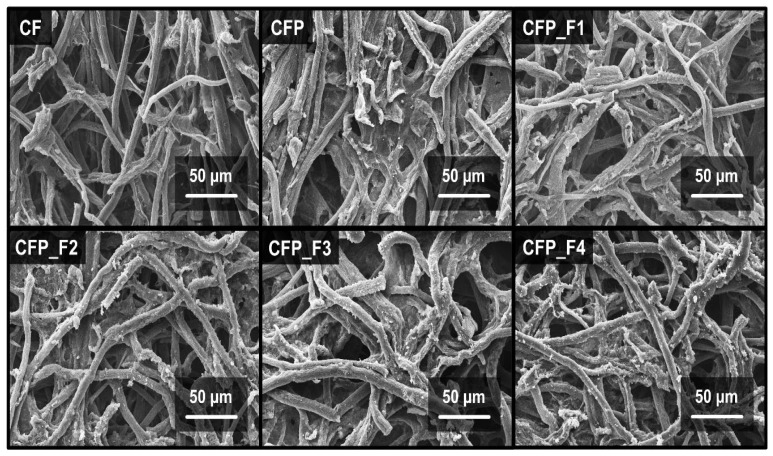
SEM images of CF, CFP, and CFP_F1-F4 samples.

SEM images in Figure 2 reveal surface morphologies of all CF paper specimens. The fibers structures with large diameters decorated with nanoparticles were observed in the CF_F1-F4 specimens, while no nanoparticles were observed in the pristine CF and CF paper. Increasing numbers of nanoparticles were detected in the CF papers with higher Fe(II/III) precursors concentrations. This is consistent with the EDX results indicating that the presence of Fe_3_O_4_ nanoparticles increased with increasing Fe(II/III) precursors concentration, as evidenced by the Fe/C ratio shown in Table 2. The EDX spectra of CF and all CF paper specimens are presented in Figure S1, in the supplementary material. 

**Figure 3:**
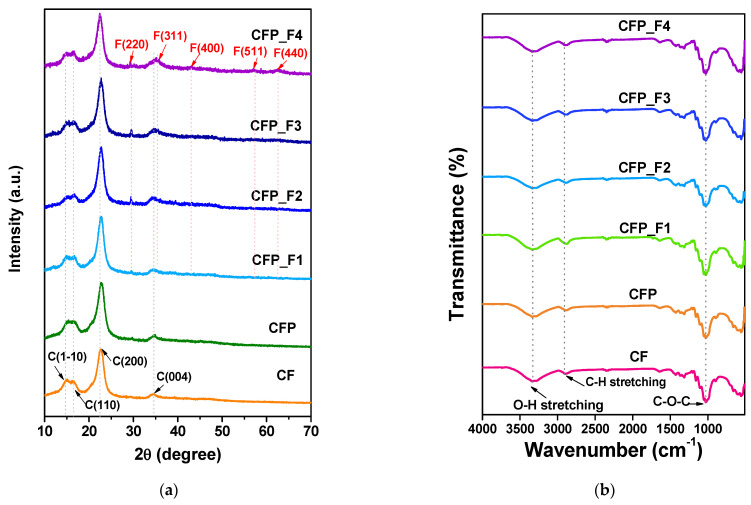
(**a**) XRD and (**b**) FTIR spectra of CF, CFP, CFP_F1-F4.

Microstructure of CF and CF–Fe_3_O_4_ papers were probed by XRD analysis, as shown in Figure 3a. The XRD patterns of all CF and CFP samples showed the diffraction peaks at 2θ of 14.9, 16.8, 22.8, and 34.4° which corresponded to the reflections from the (1-1 0), (110), (200), and (004) planes of cellulose I, respectively [28]. The additional diffraction peaks at 29.5, 35.2, 43.2, 58.9, and 62.4° were detected in CFP_F4 specimens, which are indexed to the reflections from (220), (311), (400), (511) and (440) of Fe_3_O_4_ (JCPDS No. 19-0629). Some of those peaks were also observed with relatively low intensities in CFP_F1-F3 samples. This indicated that the number of Fe_3_O_4_ nanoparticles formed was greatest in the CFP_F4 sample, which was prepared from the highest Fe (II/III) precursors. 

Chemical structures of the CF, CFP, and CFP_F1-F4 were studied by FTIR analysis. FTIR spectra of pristine CF, CFP, and CFP_F1-F4 are displayed in Figure 3b. The three main characteristic peaks of cellulose in FTIR spectra were observed in all specimens at 1030, 2895, and 3330 cm^−1^. The broad peak at 3330 cm^−1^ and the peak at 2595 cm^−1^ correspond to the stretching vibration of O–H bonds and C–H bond in polysaccharide [29,30]. The peak at 1030 is associated with the C–O–C pyranose skeleton ring vibration of cellulose molecule [31]. In addition, the peaks at 610 and 550 cm^−1^ were attributed to the C–H bend from lignin molecules [32]. However, the peak of Fe–O bonding at around 570 cm^−1^ [33] was not observed in CFP_F1-F4, which could be due to the superposition by the lignin band.

**Figure 4:**
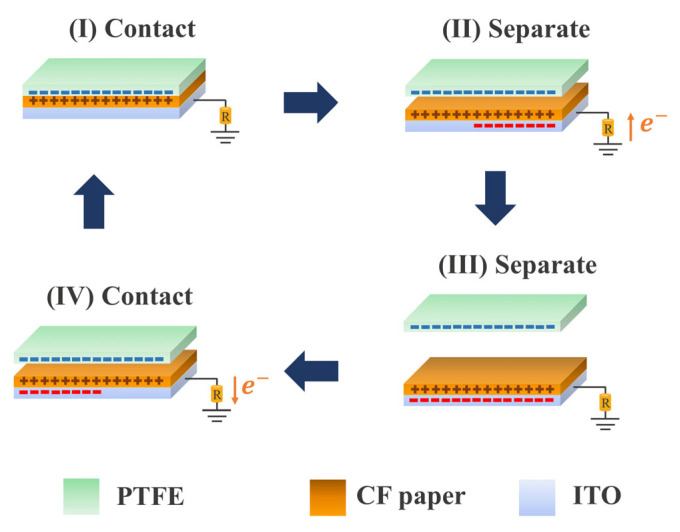
The operation mechanism of CF paper TENG in a single electrode mode.

TENG performances of the fabricated CF paper-based TENGs were tested with a Teflon sheet as a contact tribonegative material. The working mechanism of the fabricated TENG under a single electrode mode is illustrated in Figure 4. The electrical output is generated by a combination of electrification and electrostatic induction effects. When the surfaces of two different materials are contacted (state I), electrons are transferred between them due to the difference in their chemical potentials. Depending on the tendency to gain/lose electrons, surface charges or triboelectric charges with different signs are formed on the two surfaces. Since Teflon is known as the most tribonegative material, negative surface charges are formed on its surface and positive ones are formed on the CF papers. When the surfaces are separated (state II), the electrical potential is created, which induces free electrons to flow from the ground to a conductive ITO to balance the created potential. The negative current is then generated in this state. Once the surfaces are brought to contact again (state II), the electrons are flowing back since the potential disappears, generating the positive current in this state.

**Figure 5:**
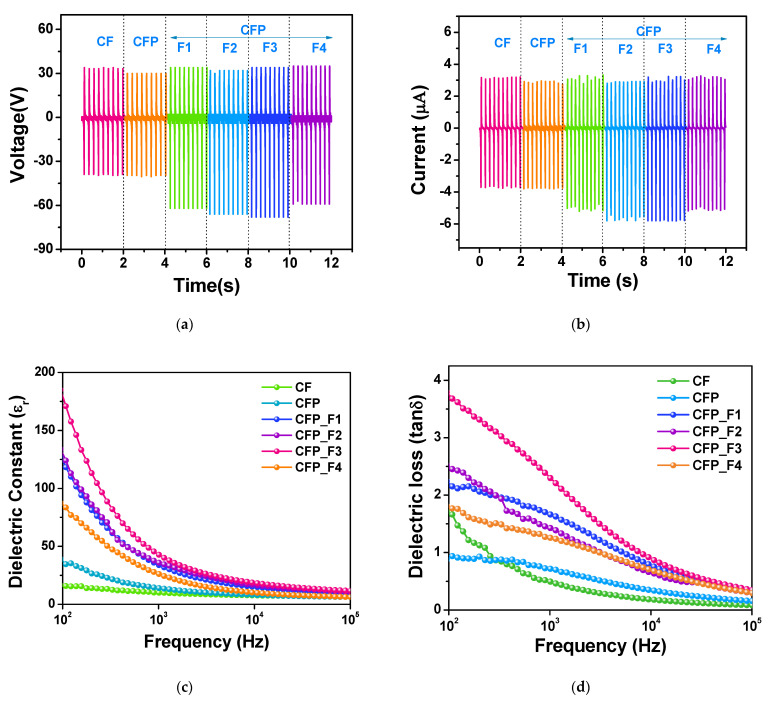
(**a**) Electrical output voltage and (**b**) current of the fabricated CF, CFP, CFP_F1-F4 TENGs under a single electrode mode at the applied impact force of 4N at 5 Hz. (**c**) Dielectric constant and (**d**) dielectric loss of CF, CFP, CFP_F1-F4 specimens.

The electrical output voltage and current measured at a constant impact frequency of 5 Hz and impact force of 4 N are presented in Figure 5a,b, respectively, and their peak-to-peak values are shown in Table 3. The output voltage and current of the Fe_3_O_4_-filled CF paper TENGs increased with increasing Fe_3_O_4_ content in the CFP_F1-3 TENGs, but reduced in the CFP_F4 one. The output voltage and current were improved from 72 V and 7.02 µA in pristine CF to 100 V and 9.2 µA in CFP_F3 TENG. To explain this result, dielectric constant and dielectric loss of the fabricated CF papers were probed and analyzed, as shown in Figure 5c,d, respectively. Dielectric constant (ε_r_) and loss tangent (tan δ) at 1 kHz of all the specimens are listed in Table 3. Dielectric constant increased to 43 in the CFP_F3, which was four and three times higher than pristine CF (ε_r_ = 10) and CFP (ε_r_ = 14), respectively. Despite having high dielectric constant, the CFP_F3 also had high dielectric loss. However, higher Fe_3_O_4_ content in the CFP_F4 did not cause the increase in dielectric loss. The dielectric loss showed the different trend from dielectric constant, which was not correlated to electrical output of the TENG. The reductions of TENG performance and dielectric constant in CFP_F4 were attributed to the high load of Fe_3_O_4_ nanoparticles over the percolation threshold, giving rise to the electrical conductivity of the composite [34]. 

**Figure 6:**
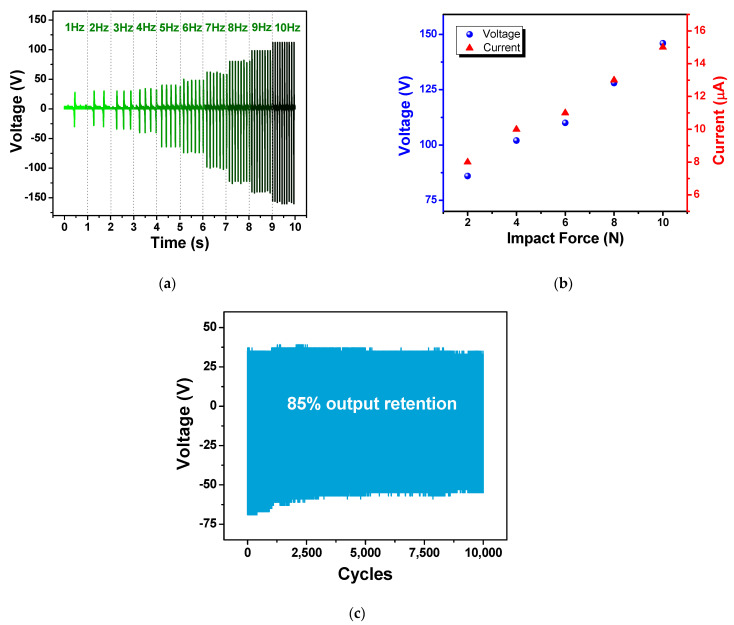
The dependence of TENG outputs on (**a**) working frequencies and (**b**) impact force of the CFP_F3 TENG; (**c**) output voltage retention of the TENG over the repeated operation of 10,000 cycles.

The dependence of electrical output on the working frequency of CFP_F3 TENG is presented in Figure 6a. Like other TENGs, output voltage increased with increasing frequency from 1–10 Hz, which reached the highest output voltage of 272 V at 10 Hz. This was ascribed to the high charge transfer rate due to the increased movement speed of the triboelectric layer [27,35,36].

The influence of impact force on electrical outputs was also probed, and the results are shown in Figure 6b. The TENG output voltage and current was found to increase with the increasing impact force from 1–10 N, and the increments of the outputs was found to be almost linearly dependent on the impact force. This was ascribed to the increased contact area due to the deformation of surface structure under the compressive force [37,38]. As the impact force increased up to 10 N, the highest voltage output of 146 V was achieved. 

The stability of electrical output was studied by measuring output voltage during repeated contact-separation motion under 4 N at 5 Hz. The result showed that the performance of the TENG fabricated from CF paper filled with Fe_3_O_4_ nanoparticles, exhibited the relatively good stability of 85% retention over 10,000 cycles at a relative humidity of 25%. 

**Figure 7:**
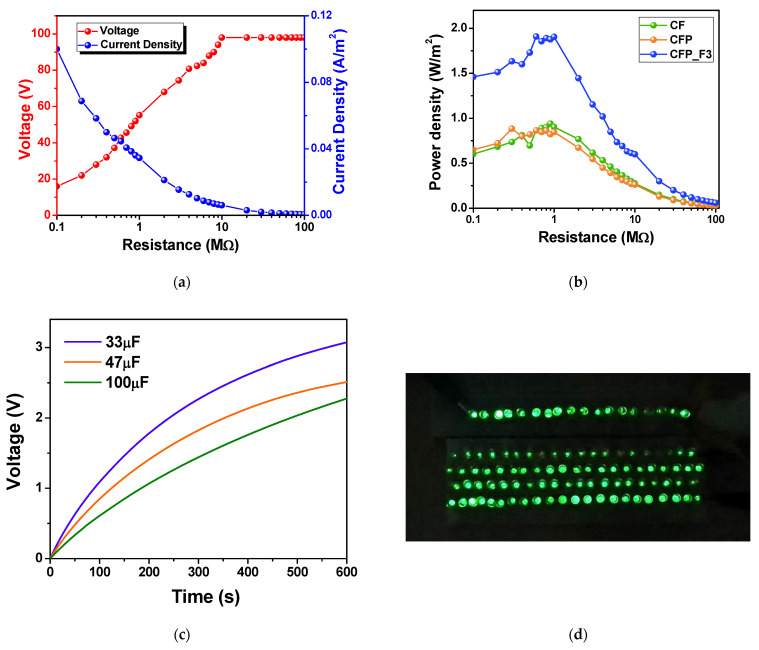
(**a**) The electrical outputs of TENG when connected to various load resistances ranging from 0.1–100 MΩ of the CFP_F3 TENG operated, and (**b**) power density of CFP_F3 TENG compared to the CF and CFP TENGs. (**c**) Voltage profile of the 33, 47, 100 µF capacitors charged by the CFP_F3 TENG. (**d**) The application of TENG to light up 100 green LEDs.

The delivered power density of the fabricated CF paper-based TENG was determined. The measured output voltage and current of the CFP_F3 TENG operated at the applied impact force of 4 N, at a working frequency of 5 Hz when connected to various load resistances ranging from 0.1–100 MΩ, are presented in Figure 7a. The plot of the calculated power densities of the CFP_F3 compared to those of the CFP and the CF TENG is displayed in Figure 7b. The highest power density of 1.9 W/m^2^ was achieved at 0.6 MΩ matched load, which was approximately double those of CF and CFP TENGs (0.9 W/m^2^). This suggested that Fe_3_O_4_ nanoparticles effectively improved the power output of the CF-based TENG. The CF paper-based TENG in this work possessed a higher power density than many previously reported cellulose paper TENGs [8], as listed in Table 4 below.

The generated electrical power from the fabricated TENG was demonstrated to charge a series of commercial capacitors, which could be used as a power source for small electronic devices. The voltage profiles of the 33, 47 and 100 µF charged by the fabricated TENG are shown in Figure 7c. The 33, 47 and 100 µF capacitors were charged to 1.5 V within 156, 220, and 320 s, respectively. The output power of TENG was also able to light up 100 green LEDs connected in series, as presented in Figure 7d. These suggest the applications of the CF paper TENGs as a power source to either instantaneously power lighting devices, or to charge an energy storage device that is subsequently used to drive micro/nanoelectronic devices.

## 4. Conclusions

The CF paper was fabricated from sugarcane leaves and used as a friction layer for TENG to convert mechanical energy into electricity. Fe_3_O_4_ nanoparticles was synthesized and filled in the CF paper to improve the energy conversion performance of the TENG. The electrical output power was found to improve with the addition of Fe_3_O_4_ nanoparticles, which was ascribed to the improved dielectric constant of the triboelectric paper. The CF-Fe_3_O_4_ paper TENG generated the highest power density of 1.9 W/m^2^, which was able to charge commercial capacitors that may serve as a power source for micro/nanoelectronic devices. The success of this work may lead to the development of natural material-based TENG with a high energy conversion performance. 

## Figures and Tables

**Table 1:** The fabrication detail and of all the fabricated specimens.

Sample Detail	Fe (II)/Fe (III) Conditions	Sample Name
CF	-	CF
CF paper + HEC	-	CFP
CF paper + Fe oxide + HEC	Fe(II) 0.05 M + Fe(III) 0.10 M	CFP_F1
CF paper + Fe oxide + HEC	Fe(II) 0.10 M + Fe(III) 0.20 M	CFP_F2
CF paper + Fe oxide + HEC	Fe(II) 0.15 M + Fe(III) 0.30 M	CFP_F3
CF paper + Fe oxide + HEC	Fe(II) 0.20 M + Fe(III) 0.40 M	CFP_F4

**Table 2:** EDX Element analysis of the CF and CFP samples.

TENG	Weight %			Fe/C	Fe/O
	C	O	Fe		
CF	55	40	-	-	-
CFP	55	40	-	-	-
CFP_F1	52	29	16	0.30	0.55
CFP_F2	45	25	26	0.58	1.04
CFP_F3	44	22	31	0.70	1.41
CFP_F4	41	22	35	0.85	1.59

**Table 3:** Electrical output voltage (*V_pp_*) and current (*I_pp_*) of CF, CFP_F1–F4 TENGs.

TENG	*V_pp_* (V)	*I_pp_* (µA)	εr	*tan δ*
CF	74	6.9	10	0.49
CFP	70	6.7	14	0.71
CFP_F1	96	8.5	34	1.66
CFP_F2	98	8.7	36	1.42
CFP_F3	102	9.0	43	2.29
CFP_F4	94	8.4	26	1.25

**Table 4:** Power densities of the fabricated CF-Fe_3_O_4_ paper TENG in this work compared to previously reported paper-based TENGs.

Paper-Based TENGs	Power Density (W/m^2^)	Ref.
CF–Fe_3_O_4_ paper	1.9	This work
Cellulose paper- BaTiO_3_	0.088	[10]
Crepe cellulose paper/nitrocellulose paper	16.1	[11]
PPy-cellulose paper/nitrocellulose	0.83	[39]
Printer paper	0.285	[40]
Printer paper	2.95	[41]
Printer paper	0.14	[42]
Cellulose nanofiber-natural rubber-activated carbon	2.74	[27]
Regenerated cellulose paper	307	[9]

## Data Availability

Not applicable.

## Supplementary Materials

The following supporting information can be downloaded at: https://www.mdpi.com/article/10.3390/polym15010094/s1, Figure S1: EDX spectra of CF, CFP, CFP_F1-F4; Figure S2: The magnified XRD patterns at 2θ in the range of 30–40°.
