# Supplementary material for: Fe3O4-Filled Cellulose Paper for Triboelectric Nanogenerator Application
Source: Polymers (Basel). 2022 Dec 26;15(1):94. doi: 10.3390/polym15010094 (PMC9824807; doi:10.3390/polym15010094)
Supplement: Supplementary file 1 [file polymers-15-00094-s001.zip › polymers-2078900-supplementary.pdf]

Supplementary materials

Fe_3O_4–Filled Cellulose Paper for Triboelectric Nanogenerator Application

Wimonsiri Yamklang [1], Teerayut Prada [1], Weeraya Bunriw [1], Walailak Kaeochana [1], Viyada Harnchana [1,2,*]

[1] Department of Physics, Khon Kaen University, Khon Kaen 40002, Thailand; wimonsiri_ya@kkumail.com (W. Y.); teerayut.prada@kkumail.com (T.P.); weeraya_b@kkumail.com (W. B); walailakkaeochana@kkumail.com (W.K.);
[2] Institute of Nanomaterials Research and Innovation for Energy (IN-RIE), Khon Kaen University, Khon Kaen 40002, Thailand; viyada@kku.ac.th
* Correspondence: viyada@kku.ac.th

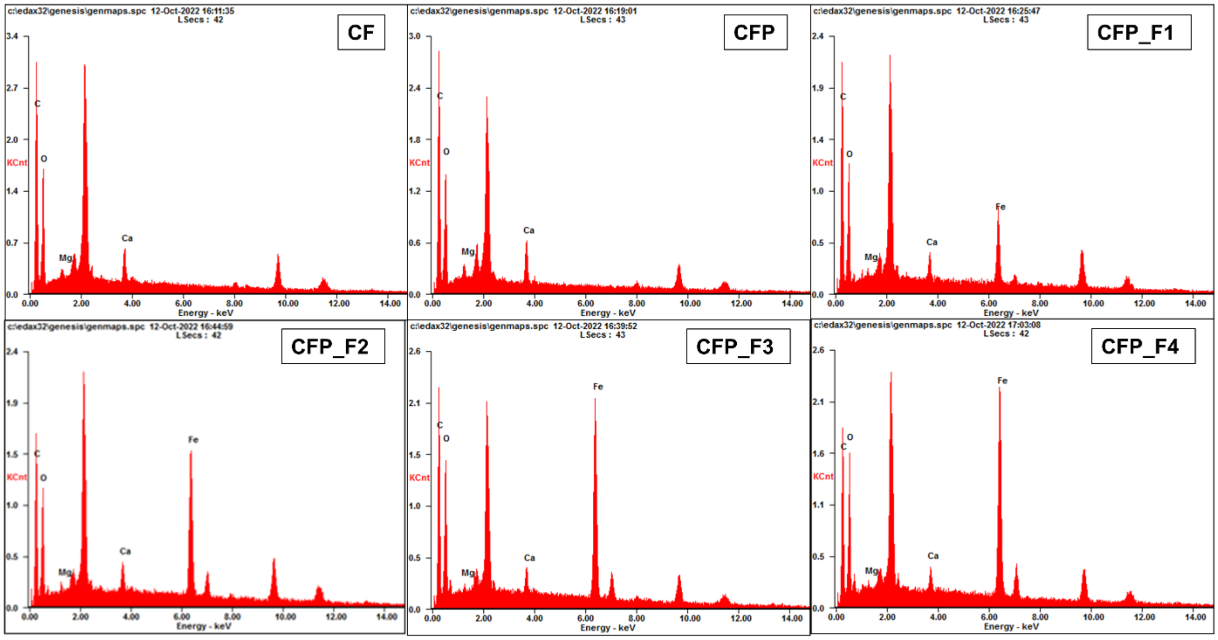

Figure S1. EDX spectra of CF, CFP, CFP_F1-F4.

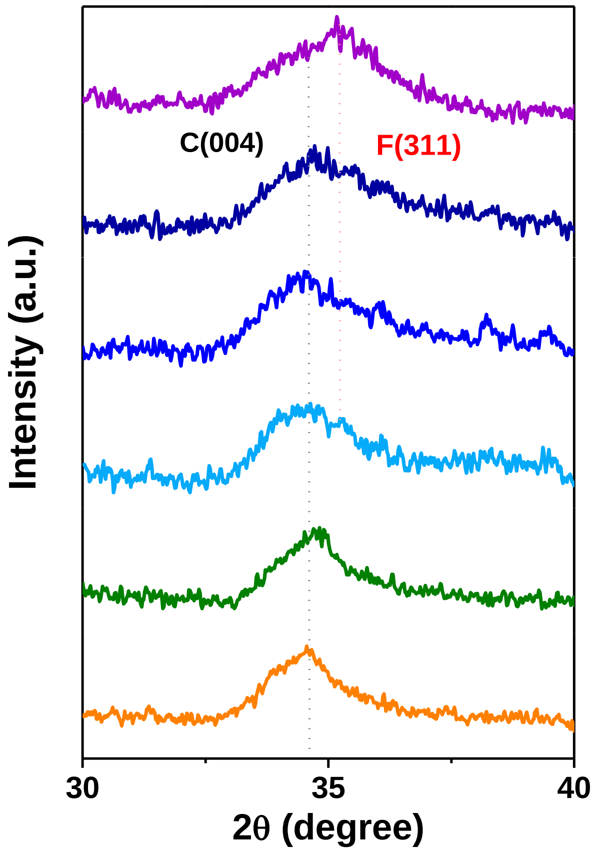

Figure S2 The magnified XRD patterns at 2θ in the range of 30-40°.
